# Effects of Activity Tracker Use With Health Professional Support or Telephone Counseling on Maintenance of Physical Activity and Health Outcomes in Older Adults: Randomized Controlled Trial

**DOI:** 10.2196/18686

**Published:** 2021-01-05

**Authors:** Katie-Jane Brickwood, Kiran D K Ahuja, Greig Watson, Jane A O'Brien, Andrew D Williams

**Affiliations:** 1 School of Health Sciences College of Health and Medicine University of Tasmania Launceston Australia; 2 School of Nursing College of Health and Medicine University of Tasmania Launceston Australia

**Keywords:** physical activity, fitness trackers, telemedicine, feedback, older adults, eHealth, mobile phone

## Abstract

**Background:**

Despite a range of efforts to increase physical activity participation in Australia, inactivity levels in older adults have remained high over recent decades, contributing to increased rates of chronic health conditions. Lifestyle interventions, including telephone counseling (TC), improve physical activity participation and associated health outcomes over the short term; however, ongoing feedback and support is required to maintain these changes. Newer technologies such as wearable activity trackers (ATs) may offer an alternative method for providing ongoing support.

**Objective:**

This study aims to investigate whether newer technologies such as wearable ATs assist in providing ongoing support to maintain physical activity levels and health outcomes.

**Methods:**

Older adults aged >60 years who had just completed a 12-week face-to-face individualized community exercise program in Tasmania, Australia, participated in the study. They were randomized to receive AT, TC, or usual care (UC). All groups received a home exercise program and an optional referral to a community-based exercise program. The AT group also received an AT and text message feedback from an accredited exercise physiologist (AEP). The TC group received phone calls from an AEP throughout the 12-month intervention. The primary outcome was daily steps measured by an ActivPAL (TM) accelerometer at baseline and at 3, 6, and 12 months. Secondary outcome measures included body composition, blood pressure, 10-time sit-to-stand (TTSTS) test, timed up and go test, and cardiorespiratory fitness. This trial was approved by the Tasmanian Health and Medical Human Research Ethics Committee (H0014713).

**Results:**

A total of 117 participants were randomized to the study (AT, n=37; TC, n=38; UC, n=42). At baseline, the participants (75/117, 64.1% female; mean age 72.4 years, SD 6.4) completed an average of 6136 steps (SD 2985) per day. Although there were no significant differences between groups, the TC and AT groups maintained daily step counts (mean difference [MD] −79 steps, 95% CI −823 to 663 steps; *P*=.81; and MD −588 steps, 95% CI −1359 to 182 steps; *P*=.09), and UC showed a reduction in daily steps (MD 981 steps, 95% CI −1668 to −294 steps; *P*=.003) during the 12-month period. Diastolic blood pressure was significantly higher after AT than after UC (MD 5.62 mm Hg, 95% CI 1.30 to 9.94 mm Hg; *P*=.01), and TTSTS was significantly slower on TC compared with UC (MD 2.36 seconds, 95% CI −0.14 to 4.87 seconds; *P*=.03).

**Conclusions:**

The use of an AT with AEP support or TC is effective at maintaining daily step count in older adults over a 12-month period, suggesting that wearable ATs are as effective as TC. Further research to investigate which option is more cost-effective would be beneficial.

**Trial Registration:**

Australian New Zealand Clinical Trial Registry ACTRN12615001104549; https://www.anzctr.org.au/Trial/Registration/TrialReview.aspx?id=369118

## Introduction

### Background

Appropriate and ongoing support is needed to assist older adults to engage in regular physical activity to minimize the functional decline and loss of independence associated with aging [1]. Structured lifestyle interventions are effective at increasing physical activity participation and improving strength and functional capacity in older adults [2,3]. Traditionally, structured lifestyle interventions use education sessions, behavior change techniques (BCTs), and self-monitoring [4]. Although these methods provide an initial increase in physical activity participation, physical activity levels tend to revert to preintervention levels once the structured intervention finishes [5,6]. As physical activity participation needs to be maintained to preserve the associated health benefits, effective strategies to assist older adults to continue to be physically active following a lifestyle intervention are required.

Several systematic reviews have assessed methods of maintaining physical activity participation [7,8]. Telephone counseling (TC) has been shown to be effective as both a booster strategy [7] and an intervention to provide ongoing support to promote habitual behavior change [8]. Evidence suggests that longer duration interventions with more regular phone calls demonstrate greater effectiveness [8]. Despite the established benefits of TC, there are significant time and resource barriers to its implementation in standard practice [9].

Activity trackers (ATs) provide consumers with the ability to objectively monitor and receive feedback relating to daily physical activity and can provide health professionals with an objective measure, allowing for the provision of targeted feedback and ongoing support [10]. Furthermore, ATs may offer a less resource-intensive alternative to TC. The use of an AT, particularly when included as part of a broader behavioral intervention, has been shown to be effective at increasing physical activity levels in different populations [11]. There is a paucity of long-term interventions (>6 months) in relation to older adults. In addition, few studies have compared the use of an AT as a stand-alone intervention compared with other established behavioral or lifestyle interventions [12,13].

### Objectives

Consequently, this study aimed to examine the effects of a commercially available AT with usual care (UC) and an established method of postintervention follow-up (TC) to assist older adults in maintaining their daily step count over a 12-month period. It was hypothesized that older adults in both the AT and TC groups would maintain physical activity levels over the 12-month intervention compared with those in the UC group.

## Methods

The study was a three-arm, 12-month randomized controlled trial (RCT) investigating the use of an AT, TC, and UC in the maintenance of physical activity levels and health outcomes in older adults. The study was approved by the Tasmanian Health and Medical Human Research Ethics Committee (H0014713) and was registered with the Australian New Zealand Clinical Trials Registry (ACTRN12615001104549). This study was reported in accordance with the CONSORT-EHEALTH (Consolidated Standards of Reporting Trials of Electronic and Mobile HEalth Applications and onLine TeleHealth) checklist [14].

### Recruitment and Randomization

To be eligible, participants had to have completed the 12-week Strength2Strength (S2S) Tasmania Exercise Treatment Initiative, be above the age of 60 years, and have or be at risk of developing a chronic medical condition. The S2S program was a community-based 12-week exercise and education program led by accredited exercise physiologists (AEPs) in Tasmania, Australia. The S2S program provided participants with individually tailored exercise programs to suit their health conditions and goals. The primary focus of the exercise program was strength-based exercises; however, a range of cardiovascular, strength, and balance exercises were included. Participants’ exercise prescriptions were reviewed weekly and progressed as required. In addition to attending the S2S program, participants were encouraged to complete an adapted version of their exercises for the home environment at least twice a week.

**Figure 1:**
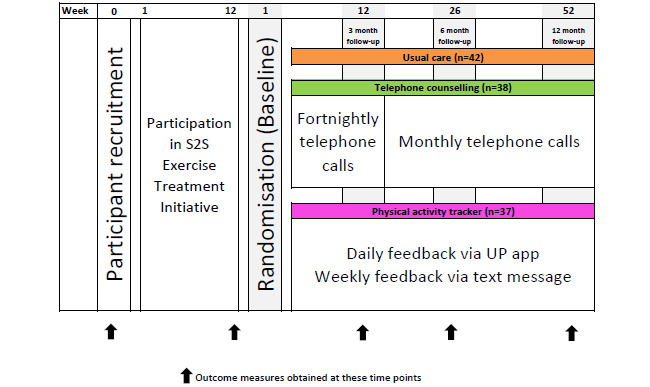
Study design showing time points for recruitment, randomization, and all data collection. All intervention groups included usual care. UC: usual care.

The RCT was a community intervention, with assessments conducted at the University of Tasmania exercise clinic. Participants were excluded from the study if they chose not to participate in the S2S program, had an unstable medical condition that prevented them from participating in regular physical activity, had a neurological condition, or had a limited understanding of English, which prevented them from meeting the self-reporting requirements of the study. All outcome measures were collected at the start and the end of the S2S program and at the 3-, 6-, and 12-month follow-up (Figure 1). Data collected at the start of the S2S program were not included in this analysis, as they were collected to inform a health economic analysis that will be reported elsewhere. Additional details regarding the S2S initiative and the design of the larger study have been published elsewhere as a protocol paper [15].

All participants were provided with information regarding the study at the beginning of the S2S program. The lead researcher answered questions and completed the consent process with interested participants. Randomization was performed using computer-generated blocks of 15 by a third person not directly involved in the study and was recorded in sealed opaque envelopes with envelopes opened sequentially at the end of the S2S program to reveal the intervention allocation. Participants from the same household were randomized to the same intervention group to maintain intervention integrity (a total of 16 participants). Participants were asked to avoid using physical activity monitoring devices other than those directly provided by researchers for the duration of the study.

### Intervention Groups

The UC group received standard care, which included the provision of an individualized home-based exercise program. The home program included similar exercises to those prescribed during the S2S program but modified for the home environment and for any equipment that was available to the participant. An optional referral to a range of community-based physical activity programs was also offered.

In addition to receiving UC, participants randomized to AT were provided with a Jawbone UP24 (TM; Jawbone, Inc) AT and ZTE (TM) mobile device and data plan. Participants who already had a compatible smartphone could choose to use the mobile device provided or their own device. No reimbursement for data costs was provided to those who chose to use their own smartphone. The device was worn on the nondominant wrist for the duration of the 12-month intervention. Participants were provided with an individual information session on how to use, pair, and charge the device at the time of randomization to the AT group. Written instructions and telephone support were provided to troubleshoot any technical issues. A separate Jawbone account was created for each participant, allowing the AEP to remotely access each participant’s daily step data. The Jawbone UP24 (TM) was paired to synchronize with the Jawbone UP (TM) app on the mobile device. A daily step goal was individually prescribed for each participant based on their physical function and current level of physical activity. This daily step goal was programmed into the UP (TM) app at the time of randomization, with automated feedback provided by the tracker and app based on this daily step goal. Participants were asked to synchronize the tracker with the UP (TM) app at the end of each day but could check the progress toward their daily step goal as desired. In addition to daily feedback available through the UP (TM) app, participants received weekly, personalized text messages from an AEP. The text message contained feedback related to average daily steps and a comparison of their daily step goal with that of the previous week. The daily step goal was slightly adjusted (±200-500 steps) during the weekly text message feedback from the AEP based on the previous week’s step data. The daily step goal in the UP (TM) app however remained the same, as this could not be adjusted remotely. If participants continued to significantly underachieve or exceed their initial daily step goal, the UP (TM) app was adjusted during their 3-, 6-, or 12-month assessment. An example of the text messages sent to participants by the AEP is provided in Multimedia Appendix 1.

Participants randomized to the TC group received UC and a physical activity counseling phone call once a fortnight for the first 3 months and once a month for the remaining 9 months of the intervention. Phone calls were delivered by an AEP experienced in motivational interviewing techniques [16], following set protocols to determine the participants’ self-reported physical activity levels. The protocol allowed the AEP to offer tailored support and advice regarding exercise prescription and modification and assist participants to identify and address any barriers limiting their physical activity participation [15]. Participants were asked to self-report activity levels and compliance with their home-based exercise program and to identify any issues preventing them from being physically active. Constructs from the Social Cognitive Theory [17], including the use of goal setting and self-monitoring, the provision of feedback, and motivational interviewing techniques (eg, affirming, reflective listening, summarizing, and informing and advising) to improve self-efficacy were used during phone calls. Participants in the TC group were asked to refrain from using a wearable AT for the duration of the intervention.

### Outcome Measures

The primary outcome measure was physical activity participation in the form of a daily step count measured by an ActivPAL (TM) accelerometer (PAL Technologies Ltd), which has been shown to be a valid and reliable device for monitoring physical activity in older adults and individuals with an altered walking gait. The ActivPAL (TM) was enclosed in a small flexible sleeve to cover the monitor and fitted to the front of the thigh using a Tegaderm (TM) film to allow participants to perform their usual daily activities (including showering or bathing) with the device in place. The ActivPAL (TM) was worn day and night for a 7-day period with a minimum of 5 days’ worth of valid data required for inclusion at each assessment time. Data files from the ActivPAL (TM) were downloaded from the devices, and event files were created using a proprietary software (ActivPAL3, version 7.2.38; PAL Technologies). Event files were analyzed using a custom software (National Instruments Labview 2017) to determine daily step counts and total nonstepping time. Only days with full recordings were considered. Daily step counts were averaged for each assessment time point. Nonstepping time was calculated by summing the time spent lying, sitting, and standing over a 24-hour period. Self-reported physical activity (total minutes of activity and metabolic equivalent [MET] per minute per week) were obtained using the Active Australia Survey (AAS) [18]. The AAS assesses leisure time physical activity and includes the number of sessions and total time spent in planned walking, vigorous-intensity gardening or housework, and planned moderate- or vigorous-intensity physical activity. The AAS has been demonstrated to be valid in community-dwelling older adults [19]. The secondary outcomes included health risk factors, functional measures, and quality of life. Health risk factors were measured using standardized protocols and included body weight, BMI, and systolic blood pressure (SBP) and diastolic blood pressure (DBP) [15]. Body fat percentage (BF%) and lean mass (LM) were assessed using bioimpedance analysis scales [20]. LM was reported in kilograms rather than percentage to provide an absolute measure of LM, which may change relative to other body composition factors. The 10-time sit-to-stand (TTSTS) test was used to measure functional lower body strength [21] and the timed up and go (TUAG) test was used to assess dynamic balance and mobility [22], both of which relate to the ability to perform activities of daily living. The 6-min walk test (6MWT) [23] was used to assess cardiorespiratory fitness for participants who had mobility issues, including walking with aids. This is a self-paced test, requiring participants to walk as many laps of a 10-meter course as they can in 6 min. The Modified Shuttle Walk Test (MSWT) is an externally paced incremental walking test requiring participants to walk, jog, or run laps of a 10-meter course, keeping pace with an audio recording until they are unable to maintain the required pace [24], and was used for the remainder of the participants. Quality of life was assessed using the Short-Form 36-Item Health Survey (SF-36) [25]. Participants’ health conditions were provided by the referring practitioner upon referral to the S2S program and checked with the participant during their initial assessment before commencing the S2S program. For ease of reporting, health conditions were categorized as follows: cardiovascular, metabolic, musculoskeletal, pulmonary, cancer, and mental health conditions. The wear time of the AT was calculated as the total number of days in which the tracker was worn divided by the total number of available days (365 days). Nonwear days were defined as days in which zero steps were recorded.

### Power Calculation

During the 12-week S2S intervention, participants reported a mean increase of 450 MET minutes of physical activity per week, with an SD of 1.5 times the change. The sample size was calculated on a predicted maintenance of 100% of additional physical activity in the AT and TC groups and a decrease of 50% of additional physical activity in the UC group over the 12-month intervention. STATA 12 (Stata Corp) was used to calculate the sample size on the basis of a mean difference (MD) of 225 MET minutes per week with an SD of the change of 350 MET minutes per week, a power of 80%, and an α level of .05. This indicated a required sample size of 38 participants per group. To allow for withdrawals, 50 participants per group were recruited.

### Statistical Analysis

All data were analyzed using STATA version 13.1 (StataCorp LLC) and graphically represented using GraphPad Prism (version 7.00 for Windows, GraphPad Software). Comparisons between the 3 interventions (as change from baseline) were made using mixed effects, repeated measures linear regression and replicated with ordered logistic regression adjusted for repeated measures because the assumptions of linear regression were not met for most variables. *P* values for comparison between groups were adjusted with Holm test for multiple comparisons. Statistical analysis was first conducted by completing the missing values through the last observation carried forward (LOCF) technique [15]. In addition, intention-to-treat (leaving missing values as blank) and per-protocol (only for people who completed all assessment time points) analyses were conducted. Intention-to-treat and LOCF included all 117 participants. The per-protocol analysis included 75 participants (UC=26, TC=25, and AT=24). The results are presented from mixed effects models in intention-to-treat analyses because it adjusts the maximum likelihood estimates based on the missing data and provides a more powerful analysis without ad hoc imputations with the LOCF analysis. This technique also retains the sample size compared with the per-protocol analysis. For ease of understanding, results comparing the 3 interventions are shown as MD and 95% CI from mixed effects, repeated measures linear regression but with *P* values obtained from logistic regression analyses adjusted for multiple comparisons.

## Results

**Figure 2:**
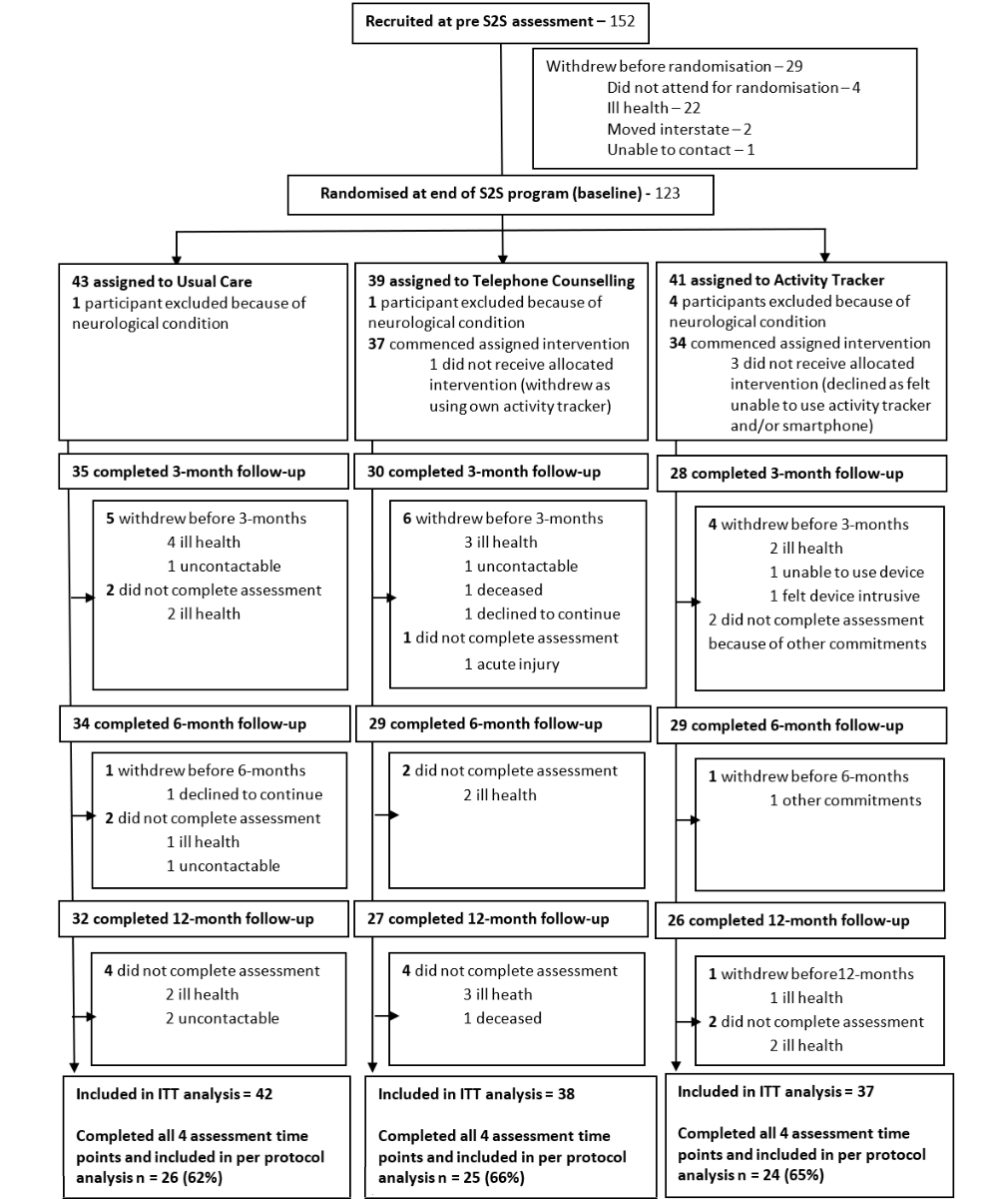
CONSORT (Consolidated Standards of Reporting Trials) diagram. ITT: Intention to Treat; S2S: Strength2Strength.

Between September 2014 and June 2016, 152 people consented to participate when starting the S2S program, with 117 randomized to one of the study intervention groups on completion of the S2S program. The average age of the participants was 72.4 years (SD 6.5; range 60.3-88.7 years). Figure 2 shows the progression of the participants through the trial. Baseline characteristics are presented in Table 1.

**Table 1:** Baseline (end of the Strength2Strength program) characteristics of randomized participants.

Outcome measure			Usual care group (n=42)		Telephone counseling group (n=38)		Activity tracker group (n=37)		Total (N=117)
Age (years), mean (SD)			71.9 (6.0)		72.8 (7)		72.3 (7)		72.4 (6.5)
**Gender, n (%)**									
	Female	29 (69)		22 (58)		24 (65)		75 (64.1)	
	Male	13 (31)		16 (42)		13 (35)		42 (35.9)	
**Physical activity, mean (SD)**									
	Steps	6590 (2908)		4996 (2533)		6764 (3244)		6136 (2985)	
	Nonstepping time (minutes per 24 hours)	1073 (109)		1099 (138)		1093 (107)		1088 (118)	
	Self-reported activity (minutes)	200 (279)		176 (187)		174 (252)		183 (239)	
	Self-reported activity (metabolic equivalents per minute)	730 (994)		608 (680)		618 (892)		652 (855)	
**Body composition, mean (SD)**									
	Weight (kg)	84.5 (23.1)		84.6 (22.6)		83.6 (19)		84.2 (21.5)	
	BMI (kg/m^2^)	31.5 (7.8)		31.2 (8.2)		30.2 (6.1)		31.0 (7.4)	
	Body fat (%)	37.9 (9.5)		35.3 (10.2)		36.9 (9.4)		36.7 (9.6)	
	Muscle mass (kg)	47.5 (10.6)		49.9 (11.1)		48.6 (9.1)		48.6 (10.3)	
**Blood pressure, mean (SD)**									
	Systolic blood pressure (mm Hg)	131 (16)		128 (20)		129 (13)		130 (17)	
	Diastolic blood pressure (mm Hg)	76 (9)		77 (10)		76 (8)		76 (9)	
**Physical function, mean (SD)**									
	Ten-time sit-to-stand (seconds)	21.7 (7.1)		21.5 (6.8)		23.7 (8.4)		22.2 (7.4)	
	Timed up and go (seconds)	7.2 (2.9)		7.5 (2.9)		7.8 (3.1)		7.5 (2.9)	
**Quality of life, mean (SD)**									
	SF-36^a^ physical health summary score	52 (22)		55 (20)		55 (20)		54 (21)	
	SF-36 mental health summary score	64 (20)		68 (16)		68 (18)		67 (18)	
**Health conditions, n (%)**									
	Cardiovascular disease	25 (59)		21 (55)		21 (57)		67 (57)	
	Metabolic disease	26 (62)		14 (37)		16 (43)		56 (48)	
	Musculoskeletal conditions	25 (59)		23 (60)		30 (81)		79 (67)	
	Pulmonary conditions	7 (17)		9 (24)		6 (16)		22 (19)	
	Cancer	8 (19)		9 (24)		4 (11)		21 (18)	
	Mental health conditions	4 (9)		9 (24)		5 (13)		18 (15)	
	≥2 chronic conditions	29 (69)		28 (74)		27 (73)		84 (72)	
	Gait aid	4 (9)		4 (10)		6 (16)		14 (12)	

^a^SF-36: Short-Form 36-Item Health Survey.

Participants in the AT group wore the AT for an average of 84% of the available days (306 out of 365 days). The number of nonwear days ranged from 1 to 164 days. The reasons for not wearing the band included forgetting to put the band on (all participants on at least one occasion), technical issues (n=20), illness (n=8), and holidays (n=5). Participants in the TC group received an average of 12 (SD 2) phone calls. Phone calls lasted an average of 11.1 min. The primary reason for missed phone calls was due to participants being away on holidays (n=5).

**Figure 3:**
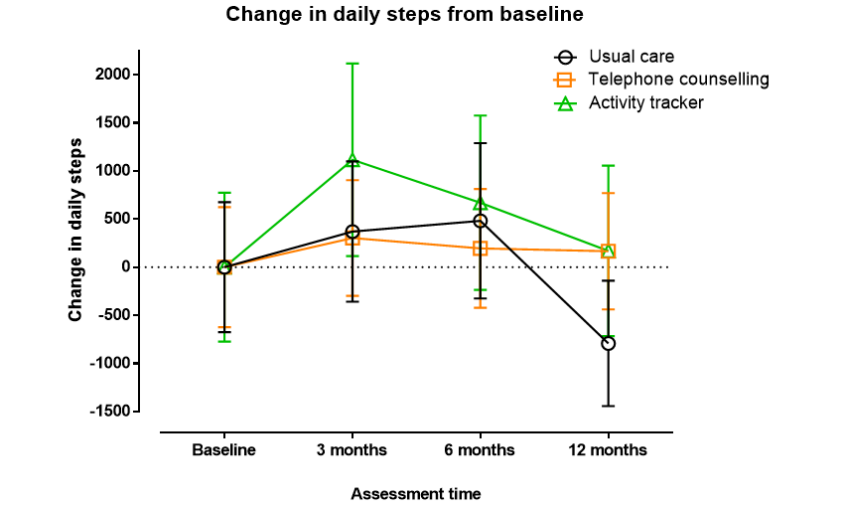
Change in daily steps with standard error of the mean between randomization and 12-month follow-up.

Over the 12-month intervention period, the UC group showed a significant reduction in the daily step count (MD 981 steps, 95% CI −1668 to −294 steps; *P*=.005). In contrast, step counts for the TC and AT groups did not change (MD −79 steps, 95% CI −823 to 663 steps; *P*=.81; and MD −588 steps, 95% CI −1359 to 182 steps; *P*=.09, respectively). There were no significant differences in changes in daily step counts between any of the groups over the 12-month intervention (*P*≥.14; Figure 3).

Objectively measured nonstepping time did not change over the 12-month period, and no differences were observed among the 3 groups between baseline and 12 months. Self-reported physical activity levels also did not change over the 12-month period, and there were no differences among the 3 groups at 12 months (Table 2).

No changes in body weight were observed over the 12-month period; however, there was a mean increase in BF% observed in both the TC and AT groups (MD 1.51%, 95% CI 0.43%-2.58%; *P*=.006; and MD 1.89%, 95% CI 0.82%-2.97%; *P*=.001, respectively). For LM, no change was observed between baseline and 12 months in the AT and TC groups, whereas the UC group showed a significant reduction in LM over the 12-month period (MD −1.13 kg, 95% CI −2.26 to −0.01 kg; *P*=.05).

DBP was significantly reduced over the 12-month period in the UC group (MD −4.10 mm Hg, 95% CI −7.02 to −1.18 mm Hg; *P*=.02), whereas no changes were observed in the TC and AT groups. SBP remained unchanged during the 12-month intervention period, and no differences were observed between the 3 groups. Therefore, there was a significant reduction in DBP in the UC group when compared with the AT group (MD 5.62 mm Hg, 95% CI 1.30 to 9.94 mm Hg; *P*=.01). No other between-group differences for DBP were observed between baseline and 12 months.

The UC group was the only group to demonstrate an improvement in TTSTS performance during the 12-month intervention period (MD −1.10, 95% CI −2.80 to 0.70; *P*=.03). The UC group performed the TTSTS significantly faster than the TC group between baseline and 12 months (MD 2.36 seconds, 95% CI −0.14 to 4.87 seconds; *P*=.03). The time taken to perform the TUAG did not change over the 12-month period for any of the intervention groups nor did the groups differ over the 12-month follow-up.

Of the 117 participants, a total of 58 participants (UC=19, TC=21, and AT=18) completed the MSWT, whereas 59 (UC=22, TC=17, and AT=18) completed the 6MWT. No differences in the distance walked during either the MSWT or 6MWT were observed between the 3 intervention groups or between baseline and 12 months within any of the intervention groups.

Self-reported physical function or mental health scores as measured by the SF-36 did not change during the 12-month intervention period for the 3 intervention groups, and there was no difference between groups at 12 months.

**Table 2:** Results of the included outcome measures at baseline and at 3, 6, and 12 months with within- and between-group *P* values.

Outcome measure			Within-group changes at 12 months					Between groups versus UC^a^	Between groups versus TC^b^
			Baseline	3 months	6 months	12 months	*P* value		
**Physical activity participation**									
	**Steps per day, mean (SD)**								
		UC	6590 (2908)	7050 (3083)	6968 (3551)	5836 (2422)	*<.01* ^c^	N/A^d^	N/A
		TC	4996 (2533)	5354 (2192)	5271 (2124)	5080 (2084)	.81	.14	N/A
		AT^e^	6764 (3244)	7937 (4324)	7552 (3701)	7091 (3241)	.09	.45	.30
	**Nonstepping time (minutes per 24 hours), median (IQR)**								
		UC	1088 (797-1141)	1035 (942-1122)	1067 (961-1158)	1101 (1015-1204)	.07	N/A	N/A
		TC	1119 (1021-1203)	1108 (965-1174)	1113 (1028-1180)	1106 (1071-1224)	.28	1.00	N/A
		AT	1085 (1034-1151)	1064 (969-1139)	1046 (992-1149)	1100 (990-1158)	.29	.67	.99
	**Self-reported activity (minutes per week), median (IQR)**								
		UC	135 (50-265)	175 (106-330)	155 (75-255)	137 (30-230)	.27	N/A	N/A
		TC	132.5 (50-400)	170 (120-260)	108.5 (40-202)	120 (60-290)	.28	.95	N/A
		AT	270 (112.5-489)	210 (140-140)	180 (95-295)	207 (90-375)	.10	1.0	1.0
	**Self-reported activity (metabolic equivalents per minute per week), median (IQR)**								
		UC	460 (170-960)	625 (109-1215)	590 (300-930)	505 (105-871)	.34	N/A	N/A
		TC	436.5(200-1330)	648 9480-965)	388 (145-765)	480 (210-1030)	.42	.98	N/A
		AT	982.5 (415-1890)	840 (525-1550)	680 (632-1047)	760 (345-1425)	.11	1.0	1.0
**Body composition**									
	**Weight (kg), mean (SD)**								
		UC	84.5 (23.1)	82.8 (24.1)	79.3 (19.8)	77.7 (17.4)	.39	N/A	N/A
		TC	84.6 (22.6)	86.2 (22.8)	86.1 (23.1)	86.6 (24.5)	.19	.24	N/A
		AT	83.6 (19)	85.1 (16.6)	85.7 (17.6)	84.7 (17.2)	.61	.83	.19
	**BMI (kg/m^2^), mean (SD)**								
		UC	31.5 (7.8)	31.2 (7.7)	30.1 (6.7)	29.9 (6.5)	.60	N/A	N/A
		TC	31.2 (8.2)	31.7 (7.8)	31.8 (8.8)	31.5 (8.9)	.93	1.0	N/A
		AT	30.2 (6.1)	30.9 (5.7)	30.8 (6.0)	30.5 (5.9)	.47	.87	1.0
	**Body fat (%), median (IQR)**								
		UC	40 (31.9-46.7)	39.9 (30-47.3)	39 (32.1-46.8)	41.8 (33.5-47.7)	.08	N/A	N/A
		TC	37.3 (27.2-44.1)	40.3 (28.3-43)	40.4 (26.6-44.6)	40.7 (30-47.1)	*<.01* ^c^	.39	N/A
		AT	38 (30.4-44.4)	38.1 (31.8-44.3)	39.9 (29.4-44.4)	39.6 (32.5-44.2)	*<.01* ^c^	.61	.95
	**Lean mass (kg), mean (SD)**								
		UC	47.5 (10.6)	45.9 (10.3)	44.9 (9.2)	43.1 (6.2)	*.05* ^c^	N/A	N/A
		TC	49.9 (11.1)	50.9 (11.1)	50.2 (10.3)	49.9 (10.8)	.36	.53	N/A
		AT	48.6 (9.1)	49.6 (9.2)	50.7 (9.5)	49.3 (8.6)	.84	.49	.62
**Blood pressure**									
	**Systolic blood pressure (mm Hg), median (IQR)**								
		UC	130 (122-140)	137 (124-140)	129 (120-135)	130 (125-140)	.71	N/A	N/A
		TC	130(120-140)	129 (120-137)	128 (123-144)	130 (122-135)	.95	.84	N/A
		AT	128 (120-138)	128 (120-139)	130 (122-135)	129 (125-140)	.80	.94	.90
	**Diastolic blood pressure (mm Hg), median (IQR)**								
		UC	74(70-80)	75 (70-80)	73 (65-79)	70 (70-75)	*.02* ^c^	N/A	N/A
		TC	76(70-83)	78 (70-84)	75 (70-80)	75 (70-80)	.35	.36	N/A
		AT	78(70-80)	76 (73-80)	78 (70-80)	80 (74-80)	.11	.01^c^	.15
**Physical function**									
	**Ten-time sit-to-stand (seconds), median (IQR)**								
		UC	20.3 (16.8-24.1)	18.9 (16.4-22.6)	19.3 (15.9-24.5)	18.3 (14.9-24.0)	*.03* ^c^	N/A	N/A
		TC	20.9 (15.9-23.8)	20.3 (18.5-24.4)	20.7 (17.4-24.5)	20.5 (16.5-22.7)	.18	*.02* ^c^	N/A
		AT	21.3 (18.6-27.9)	19.7 (14.9-27.3)	19.7 (16.2-28.0)	21.7 (16.7-28.3)	.97	.14	.38
	**Timed up and go (seconds), mean (SD)**								
		UC	7.2 (2.9)	6.9 (2.6)	7.2 (2.5)	6.8 (2.6)	.71	N/A	N/A
		TC	7.5 (2.9)	7.2 (2.2)	7.3 (2.4)	7.3 (2.3)	.17	.83	N/A
		AT	7.8 (3.1)	7.9 (3.6)	7.5 (3.0)	7.9 (3.2)	.21	.47	.93
	**6** **-min walk test (meters), median (IQR)**								
		UC	345 (299-413)	324 (272-400)	352 (300-384)	350 (335-422)	.51	N/A	N/A
		TC	298 (220-450)	336 (244-430)	338 (294-430)	358 (312-467)	.24	.38	N/A
		AT	375 (240-400)	342 (244-380)	295 (228-410)	291 (244-445)	.30	.72	.24
	**Modified shuttle walk test (meters), median (IQR)**								
		UC	460 (360-570)	520 (390-640)	530 (440-660)	450 (415-590)	.48	N/A	N/A
		TC	440 (380-540)	480 (350-570)	555 (440-600)	530 (440-590)	.31	.84	N/A
		AT	490 (390-560)	630 (520-640)	555 (480-630)	555 (450-620)	.26	1.0	.86
**Quality of life**									
	**SF-36^f^ physical health summary score, median (IQR)**								
		UC	50 (36-72)	51.5 (31.5-77)	51 (31-72)	63 (39-71)	.48	N/A	N/A
		TC	60 (38-72)	49 (68-70)	52 (41-68)	52 (43-69)	.76	.97	N/A
		AT	53 (39-73)	54.5 (40-77)	61 (36-79)	60 (47-70)	.19	.63	.51
	**SF-36 mental health summary score, median (IQR)**								
		UC	68 (53-84)	65.5 (54-83)	67 (42-82)	77 (52-84)	.76	N/A	N/A
		TC	72.5 (52-82)	73 (54-80)	69 (54-78)	77 (55-84)	.73	.96	N/A
		AT	72 (53-83)	71 (53-83)	73 (62-82)	76 (68-82)	.35	1.0	1.0

^a^UC: usual care.

^b^TC: telephone counseling.

^c^Statistically significant.

^d^N/A: not applicable.

^e^AT: activity tracker.

^f^SF-36: Short-Form 36-Item Health Survey.

The results from the LOCF and per-protocol analyses showed similar results with a few key exceptions. For the LOCF analysis, the UC group showed a significant increase in nonstepping time (*P*=.05) between baseline and 12 months, whereas the AT group showed a significant decrease in self-reported minutes of physical activity per week between baseline and 12 months (*P*=.04). This was also observed in the per-protocol analysis (self-reported minutes per week, *P*=.02; self-reported MET per minute per week, *P*=.01). The previously observed significant decrease in LM and time taken to perform the TTSTS between baseline and 12 months for the UC group was found to be no longer significant following the LOCF analysis (*P*=.09 and *P*=.07, respectively). Following the per-protocol analysis, a significant decrease in body weight was observed in the UC group (*P*=.05), and DBP in the AT group was found to differ significantly from the TC group (*P*=.05) in addition to the UC group, which was observed in the intention-to-treat analysis.

## Discussion

### Principal Findings

This study aimed to investigate how wearable ATs may assist in providing ongoing support to maintain physical activity levels and health outcomes in older adults compared with TC and UC over a 12-month intervention. We found that both ATs and TC were similarly effective at successfully maintaining daily step count over the 12-month intervention. The UC group maintained daily step count throughout the 9 months; however, a significant reduction in daily steps was observed at 12 months. Although previous research has suggested that the use of ATs either as part of a broader intervention or as a stand-alone intervention can significantly improve daily step counts [11], this is the first study to investigate the use of ATs to assist in the maintenance of physical activity over a 12-month period following a structured lifestyle intervention. Previous research has indicated that wearable ATs can help maintain physical activity participation (in cancer survivors) in the 3 months following a lifestyle intervention [26]. Using step count as a proxy for physical activity, our data demonstrate that, in older adults, this benefit can last at least a year and is as effective as the next best alternative, TC.

Interestingly, the results of the LOCF analysis showed a significant reduction in self-reported physical activity during the 12-month intervention in the AT group, reporting a mean decrease of 100 min of activity per week. The AT group was the only intervention group to consistently self-report performing at least 150 min of activity throughout the 12-month intervention and was performing between 70 and 87 min more of physical activity than both the UC and TC groups at 12 months. As this is approximately 50% of the recommended levels of physical activity, the AT group exceeded the minimally important clinical difference when compared with the TC and UC groups [27]. This suggests that even though a significant decrease in self-reported physical activity was observed in the AT group, it is unlikely that it had any significant implications for participants because of their overall higher levels of activity. It is also important to consider that results from the LOCF analysis are unlikely to reflect the true results of the study [28], particularly because of the high number of participants (15/113, 13.2%) who withdrew before completing the 3-month assessment. As noted in the *Methods* section, the LOCF analysis was completed as it was specified in the published protocol for this study [15] before the withdrawal of these participants.

Although overall body weight did not change for any of the included intervention groups, some changes in body composition were observed. An increase in BF% was observed in all intervention groups but was significant only in the TC and AT groups. Although elevated BF increases the risk of cardiovascular disease [29] and is associated with increased mortality [30], preservation of physical activity levels reduces mortality risk, even in individuals with elevated BF [31]. Despite the significant increase in BF%, this equated to a mean increase of only 0.3 kg in fat mass for both the AT and TC groups and therefore unlikely to place them at greater risk of chronic health conditions. In addition, the UC group showed a significant decrease in LM, with a reduction of almost 10% in LM between baseline and 12 months. The few previous studies that examined the effects of AT use on body composition and have included LM either reported decreases in LM across all groups potentially due to the incorporation of dietary intake restrictions or reported no change [32,33]. A key difference between this study and previous studies, which may potentially explain why LM was maintained in the AT and TC groups, is that the participants of this study were older and less healthy and therefore at an increased risk of developing sarcopenia. We speculate that the additional physical activity performed by the AT and TC groups helped to preserve their LM and subsequently assisted in delaying obligatory age-related sarcopenia, as has been shown previously in older adults who exercise [34]. In the long term, this preservation of LM could also subsequently reduce functional decline and mortality risk [35,36].

Although unexpected, a significant reduction in DBP and an improvement in physical function as measured by TTSTS was observed in the UC group, with significant differences also observed between the UC and AT groups for DBP and the UC and TC groups for TTSTS. The reason for the decrease in DBP observed in the UC group and the observed difference between the UC and AT groups are potentially related to the 6.8 kg weight loss in the UC group. Although this finding was not statistically significant, it did exceed the minimum clinically important difference for weight loss [37]. As the association between weight loss and reduction in blood pressure is well documented in individuals both with and without hypertension [38], this may have influenced the observed reduction in DBP in the UC group. In relation to the observed reduction in TTSTS time at 12 months in the UC group and differences between the UC and TC groups for the time taken to perform a TTSTS, the reasons are less clear. It has been suggested that weight loss alone can result in significant improvements in physical performance in frail, obese older adults [39]; however, because of the significant loss in LM observed, this may not fully explain the results observed. For a 5-time sit-to-stand test, the minimum clinically important difference is 1.7 seconds [40]. As the MD between the UC and TC groups was 2.3 seconds (95% CI −0.1 to 4.8 seconds) for the TTSTS, it is unclear if this would have a clinically meaningful effect. Furthermore, when analyzed using the LOCF analysis, a significant reduction in TTSTS time in the UC group was no longer observed. However, the difference between the UC and TC groups remained.

As ATs and TC were similarly effective in this study, the advantages and disadvantages of both methods should be considered to help determine if one is more suitable than the other and has more potential to encourage increases or maintenance in physical activity. ATs have several potential benefits compared with TC. An AT allows individuals to self-monitor physical activity, which can improve physical activity participation [41]. Furthermore, sharing objectively measured data assists health care professionals in providing tailored feedback, which improves self-management [42]. In comparison, TC can be time intensive and resource intensive [9] and typically relies on subjective self-reported data, which can be unreliable in older adults [43]. It is important to note that this study reported that telephone calls lasted an average of 11 min per call, which is significantly less than previously reported studies [8,44]. The reason for this was most likely that the telephone calls provided served as a *check-in* with BCTs used as required to assist the maintenance of activity levels rather than as an intervention aimed at increasing physical activity participation. Currently, as there are limited options available that allow the sharing of AT data with health care professionals, it is acknowledged that providing feedback based on an individual’s AT data can also be time intensive and resource intensive because of the data mining and interpretation required. Through improvements in the interoperability of proprietary and third-party mobile apps, the level of data mining would be reduced [45]. In addition, the development of a platform to specifically facilitate patient AT data transfer to health professionals would further reduce the time and resource burden of providing ongoing, tailored support.

### Strengths and Limitations

A key strength of this study was the long-term intervention period, as most previous research included interventions of no longer than 6 months. The study design also allowed for direct comparison between feedback provided by an AT with TC, an established method of providing ongoing support. Evaluating the effect of newer technologies such as ATs is important in understanding how they can be incorporated into standard clinical practice. In addition, participants were recruited from a clinical exercise program, offering a good representation of the chronic conditions present in community-dwelling older adults.

A limitation of this study potentially affecting the ability to interpret results was that the a priori sample size was not met. A total of 150 participants were recruited at the start of the S2S program; however, a number of participants did not complete the S2S program and were not randomized. Owing to the cessation of the S2S program, additional participants could not be recruited to account for the withdrawals before the intervention. Further dropouts occurred during the 12-month intervention. The primary reason for withdrawal was ill health, which is not unexpected, given that more than 70% of the study population had 2 or more chronic conditions. Overall, 5 participants withdrew from the AT group because of feeling uncomfortable using the device. Although ATs are well accepted in older adult populations [46], this highlights that ATs may not be suitable for all older adults. Another consideration relates to the frequency in which feedback was provided in each of the intervention groups. Although the AT group received weekly feedback, the TC group received fortnightly feedback for the first 3 months and monthly feedback thereafter. It is possible that more regular feedback for the TC group may have improved participant outcomes. Finally, it is important to note that the placement of the ActivPAL (TM) on the front of the thigh means that some exercises, including the upper body and seated strength exercises, would not be captured.

### Conclusions

Both TC- and AT-based interventions are effective at maintaining physical activity levels in older adults following a structured lifestyle intervention. As connected health technologies improve, ATs may provide an alternative to TC to assist older adults to remain active. The costs associated with delivering each intervention and the effects of each intervention on health utility should also be considered and investigated further.

### Practical Implications

The following practical implications arose from the study findings:

- A consumer-based wearable AT is an effective alternative to traditional TC support to assist older adults in maintaining daily step counts.
- Clinicians should consider the individual needs of patients to determine whether TC or an AT is better suited to provide ongoing feedback and support.

## Appendix

Multimedia Appendix 1 Example of weekly text messages sent to AT participants and Jawbone UP app user interface showing progress towards daily step goal.

Multimedia Appendix 2 CONSORT-eHEALTH checklist (V1.6.1).

## Abbreviations

6MWT: 6-min walk test
AAS: Active Australia Survey
AEP: accredited exercise physiologist
AT: activity tracker intervention
BCT: behavior change technique
BF: body fat
DBP: diastolic blood pressure
LM: lean mass
LOCF: last observation carried forward
MD: mean difference
MET: metabolic equivalent
MSWT: Modified Shuttle Walk Test
RCT: randomized controlled trial
S2S: Strength2Strength
SBP: systolic blood pressure
SF-36: Short-Form 36-Item Health Survey
TC: telephone counseling
TTSTS: 10-time sit-to-stand
TUAG: timed up and go
UC: usual care
