# Supplementary figures and images for: Effects of Activity Tracker Use With Health Professional Support or Telephone Counseling on Maintenance of Physical Activity and Health Outcomes in Older Adults: Randomized Controlled Trial
Source: JMIR Mhealth Uhealth. 2021 Jan 5;9(1):e18686. doi: 10.2196/18686 (PMC7815450; doi:10.2196/18686)

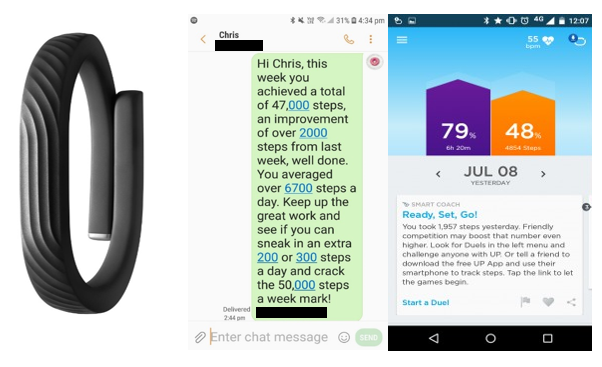

Supplement: Multimedia Appendix 1 [file mhealth_v9i1e18686_app1.png]
